# A hybrid ensemble deep learning framework with novel metaheuristic optimization for scalable malicious website detection

**DOI:** 10.1038/s41598-025-33695-z

**Published:** 2025-12-24

**Authors:** Ting Yang, JiaBao Sun

**Affiliations:** Shaoxing Institute of Technology, Shaoxing, 312000 Zhejiang China

**Keywords:** Malicious website detection, Cybersecurity, Metaheuristic optimization, Deep neural network, Cybersecurity, Engineering, Mathematics and computing

## Abstract

The rapid expansion of malicious websites poses a critical threat to online security, as conventional blacklist-based and manual inspection methods cannot keep pace with evolving attacks. In this study, we present a hybrid detection framework that integrates ensemble learning models, Random Forest, Extreme Gradient Boosting, and Light Gradient Boosting with a Deep Neural Network to distinguish malicious from benign websites accurately. The framework leverages a large-scale dataset of 63,191 URLs, combining application-layer attributes (such as URL structure, server type, and WHOIS data) with network-layer features (including TCP exchanges, DNS queries, and packet statistics). Dimensionality reduction is achieved through Principal Component Analysis, while model explainability is provided by SHapley Additive exPlanations. To enhance predictive performance, hyperparameters are tuned using two recent metaheuristic algorithms: the Weevil Damage Optimization Algorithm and the Energy Valley Optimizer. A rigorous k-fold cross-validation strategy confirms the robustness and generalization capability of the model. Experimental results demonstrate that the optimized hybrid framework surpasses individual classifiers, delivering high accuracy, strong scalability, and interpretability. This work contributes to proactive cybersecurity defenses by offering a reliable, data-driven, and explainable solution for real-time malicious website detection.

## Introduction

The WWW was a crucial innovation in the history of the Internet as it practically opened the door for anyone to access the Internet in a way that was not possible before^1^. The emergence of social networking sites and blogs has made it easier for people to share, access, and exchange ideas. The Internet was instrumental in the expansion and globalization of businesses, the only limit being the horizon they could reach via the web. Markup Language for Hypertext and Hypertext. The URL was the way browsers locate, interpret, and download any library of the online world when HTML became a tool for creating web pages in an efficient manner^2,3^. The number of web domains exploded due to the increased number of Internet users. The development of the web attracted hackers who started distributing malware and thus threatening the availability, confidentiality, and integrity of online resources^4,5^. The rise of malicious websites is steep every year, and those websites are usually created by cybercriminals to access the devices of innocent victims secretly and even to change a large number of computers into bots that can be used to perform cyberattacks on specific organizations. By misleading users through tactics that include creating websites that look like good company websites, fraudsters get data from users, infect users with malware, and make them buy fake products. In addition to fraudulent websites, there are more and more, for instance, COVID-related websites that give false information to lure users^6^. Following the uptrend in malicious URLs, researchers have introduced defense techniques based on heuristic, anomaly, and blacklisting methods. Blacklisting-based methods operate by initially compiling a list of harmful URLs and then utilizing this list to locate them. Due to the necessity for exact matching, this method is only effective against known malicious URLs and can be easily bypassed. Anomaly-based methods develop classification models for the discriminative rules or features that are used for detecting malicious URLs^7^.

The main challenge in making these methods work effectively is the problem of choosing the correct discriminative features. Heuristic methods for website scanning generate signatures of the normal assault patterns^8,9^. One major limitation of these methods is their incapacity to recognize new attack types, and hackers can easily bypass the predefined signatures^10^. As a result of these limitations, recent studies have been inclined towards ML and DL methods more than before to enhance the detection of malicious websites. Compared to conventional methods, ML and DL-based models are able to automatically extract complex patterns from large-scale URL and network traffic datasets, thereby facilitating the identification of new threats^11,12^. In a parallel manner, Patgiri et al.^13^ endeavored to build numerous detection models by implementing both supervised and unsupervised learning methods over various sampling and feature transformation settings. The selection of 34 features was the result of a study that revolved around lexical and content-based features. Only the features that were not important were removed with the help of the XGB method, whereas the feature importance was calculated by using Gini Impurity. On the basis of sampling, three different methods are chosen to deal with the unbalanced data set, i.e., Under-sampling, No-sampling, and Over-sampling. The researchers have built two models, namely SVM and AE, that are based on unsupervised learning methods. As well as that, they prepared eight different scenarios using several supervised learning algorithms such as RF, ET, GB, XGB, KNN, AB, BC, the VC, and ET. The VC was the one that gave them the highest accuracy rate of 98.03%.

Kumi et al.^14^ leveraged a dataset developed by Manjeri et al.^15^ to lay out a model for automatically classifying URLs into safe or harmful categories. They separated the dataset into 1,565 benign URLs and 216 malicious ones. The researchers applied a feature selection method named RFE as well as the SMOTE technique to oversample the data so that there would be the same number of samples for each class. They implemented SVM, DT, RF, LR, and KNN as their classification algorithms, and used accuracy, precision, and recall as their evaluation criteria. RF produced the outcomes with the highest accuracy of 96%. Not all characteristics of the lexicon, content, and network were taken into account in all research. An instance of that is the data mining technique based on CBA by McGahagan et al.^16^, which only uses the lexical and content criteria to decide whether URLs are dangerous or safe. The text that the researcher used was the dataset that came from a number of different sources. The crawling from the top sites of ALexa resulted in benign URLs from the top 500 sites. Malicious URLs came out of OpenPhish, VxVault, and URLhaus. Two components of a classifier builder, CBA-CB and CBA-RG, are the CBA. Twelve characteristics were selected through a feature selection approach. Precision, recall, accuracy, and the confusion matrix were used to gauge the suggested method. The highest performance of the CBA model was 95.83% accuracy in the classification of URLs with minimal false positive and negative rates. Urcuqui et al.^17^ also used machine learning methods to detect malicious URLs. The main goal of the study was for the authors to compare the performance of classifiers by choosing certain features with CSE versus using all the features. 20 content-based, lexical, and network-based attributes were included in the 1,781 records of the dataset utilized. The CSE method, which entails rescaling the value of features according to their informativeness and redundancy, was used for selecting features. The authors experimented with four classifiers: BayesNet, RF, KNN, and J48. The three metrics of performance were accuracy, recall, and precision. RF achieved the best results with 95.4% recall, 95% accuracy, and 95.3% precision.

While machine learning techniques have been extensively used in various studies for identifying malicious websites, a number of limitations still have not been addressed^18,19^. Large parts of existing works depend heavily on a small number of features, and most of the time, only lexical and content-based attributes are taken into account, while network-layer characteristics are ignored, which could improve detection robustness. Furthermore, most earlier methods are heavily dependent on traditional classifiers, without the integration of advanced hybrid ensemble–deep learning architectures capable of capturing complex, non-linear relationships in large-scale URL datasets. Just like in previous research, feature selection has normally been carried out by using traditional importance metrics or wrapper methods without putting much emphasis on dimensionality reduction techniques that may enhance both efficiency and generalization. The problem of model interpretability is still an issue that has not been solved, and those few attempts where researchers have used explainable AI techniques like SHAP to facilitate decision-making transparency, which is a prerequisite for the field of cybersecurity, are limited.

This research introduces a hybrid detection framework that integrates a combination of RFC, XGB, and LGBC with a DNN to unleash the power of both traditional and deep learning models. The system exploits a rich dataset of application-layer and network-layer features that enables a more comprehensive depiction of the behavior of both malicious and benign websites. To improve computational efficiency, dimensionality reduction is achieved via PCA, while SHAP is used to understand model outputs and identify the most influential features. To tune the hyperparameters, the paper uses two new metaheuristic algorithms (Weevil Damage Optimization Algorithm (WDOA) and Energy Valley Optimizer (EVO)) that have never been utilized in this area before. The robustness of the model is ensured through the k-fold cross-validation method, which ensures fair performance. Based on the experimental work, findings reveal that the devised plan outperforms in attaining higher accuracy, scalability, and interpretability as compared to other methods, thus presenting a convenient and viable approach for real-time malicious website detection.

While the WDOA and EVO are general-purpose metaheuristic techniques, their integration with a hybrid ensemble–deep learning framework for malicious website detection is novel. By jointly optimizing the hyperparameters of both ensemble and neural network models, the proposed approach enhances accuracy, generalization, and robustness beyond conventional tuning methods. This work emphasizes practical impact and explainability in cybersecurity, bridging methodological advancements with operational applicability.

### Related work

Detecting network threats and malicious web resources has been an active research area for decades. Systematic reviews of intrusion detection and related network-security research highlight the diversity of detection paradigms (signature/blacklist, anomaly detection, hybrid systems) and emphasize the importance of feature engineering, dataset heterogeneity, and performance reproducibility in operational settings^20^.

#### Classical approaches and blacklists

Early defenses against malicious URLs relied primarily on blacklists and rule-based systems that match known signatures or heuristics. These systems are lightweight and interpretable but suffer from poor coverage against zero-day and obfuscated attacks because they cannot generalize beyond previously observed samples. Machine-readable feeds (e.g., commercial threat feeds) partially mitigate this limitation but still lag behind rapidly evolving attack campaigns^21^.

#### Machine learning and deep learning for malicious URL detection

Over the past decade, research has shifted toward data-driven ML and DL techniques that use lexical, host, WHOIS, and network-level features to detect phishing and malware URLs. Surveys and recent reviews identify common strategies — feature extraction from the URL string, inclusion of contextual/network telemetry, and the use of supervised classifiers (SVM, Random Forest, XGBoost) or neural methods — and they highlight reproducibility issues driven by dataset imbalance and inconsistent preprocessing. More recent reviews also document the emergence of transformer/LLM-based defenses and the need for standardized benchmarks and open code repositories^21^.

#### Hybrid and ensemble architectures

Hybrid architectures that combine multiple classifiers or ensemble many learners have gained traction because they balance robustness, interpretability, and predictive power. Recent work demonstrates that ensembling tree-based models with neural models can improve detection rates and reduce variance compared with single-model approaches, especially when combined with careful feature engineering and remedies for class imbalance (oversampling or cost-sensitive learning). These hybrid designs are particularly useful in heterogeneous feature spaces where some features are well handled by tree models (categorical/structured) while others benefit from neural network-based representation learning^22^.

#### Metaheuristic optimization and explainability gaps

Metaheuristic algorithms (e.g., particle swarm/evolutionary methods and newer proposals such as the Energy Valley Optimizer and Weevil Damage Optimization Algorithm) have been applied to hyperparameter tuning and feature selection across many engineering domains. EVO and WDOA have shown competitive performance on benchmark optimization problems, but their application specifically to malicious-URL model tuning and to joint optimization across ensemble + deep models is relatively unexplored. Similarly, although explainability methods (SHAP, LIME) are increasingly used in security to improve analyst trust and compliance, many prior studies either omit interpretability or only report feature importance for individual models rather than for hybrid pipelines^23^.

#### Gap and the study contribution

Taken together, prior work provides strong evidence that ML/DL methods can outperform blacklist approaches on curated datasets, but three persistent gaps remain: (1) few studies jointly optimize hybrid ensemble + deep models with modern metaheuristics; (2) limited use of explainability methods across hybrid pipelines; and (3) inconsistent benchmarking across multiple datasets and real-time deployment considerations. The current study addresses these gaps by integrating RF, XGBoost, LightGBM, and a DNN into a single hybrid framework, applying EVO and WDOA for joint hyperparameter optimization, and using SHAP to provide model-level interpretability. This positions the present study as a practical and reproducible advancement in the space of explainable, high-performance malicious website detection^20^.

## Materials and methods

### Data preprocessing

#### Data description

**Fig. 1:**
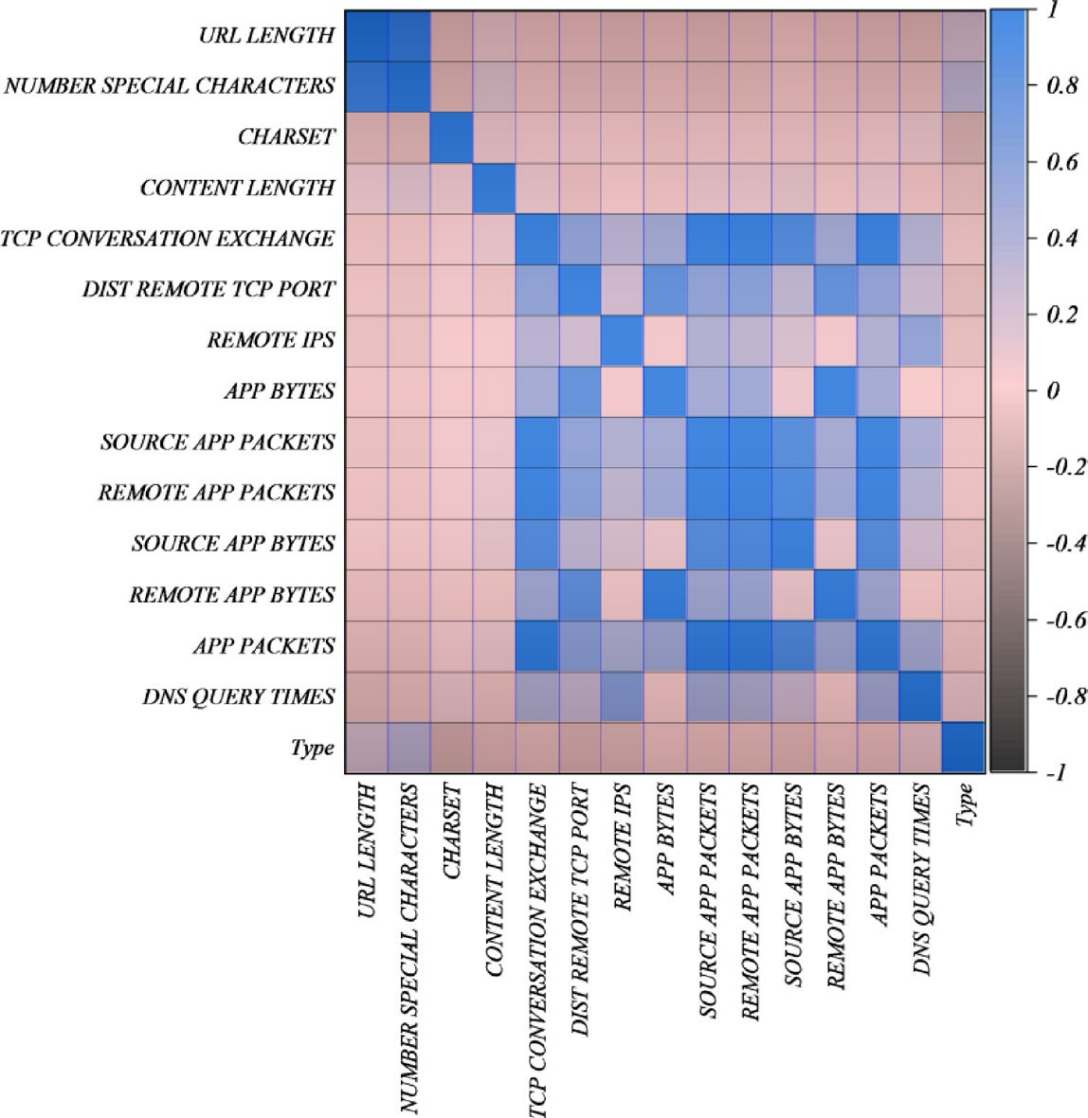
Correlation plot showing the relationships between features in the dataset.

The data for the research has been taken from a collection of harmful and normal websites that was created as part of a research project in web security, and was initially put together by Urcuqui et al.^17^ and can be accessed through this link: https://www.kaggle.com/datasets/xwolf12/malicious-and-benign-websites. The original dataset contained 530,181 records (185,181 malicious and 345,000 benign URLs), of which 63,191 valid samples were obtained after running availability checks and data cleaning. Python scripts were used for inspecting each URL to get the features of the application and network layers. Application-layer features included lexical properties (e.g., URL Length, Number Special Characters), server-related metadata (Server, Charset, Content Length), and WHOIS-based attributes (Whois Country, WhoisStatepro, Whois Regdate, Whois Updated Date). Network-layer features were obtained through a low-interaction honeypot, including TCP Conversation Exchange, Dist Remote TCP Port, Remote IPs, App Bytes, Source App Packets, Remote App Packets, Source App Bytes, Remote App Bytes, App Packets, and Dns Query Timew. The target variable TYPE indicates whether a website is malicious (1) or benign (0). To better understand the relationships among variables, a Pearson correlation analysis was performed. Figure 1 illustrates the relationship between features using a heatmap, where darker blue indicates stronger positive correlations and darker brown indicates stronger negative correlations. The study shows that several network features, like App Bytes, Source App Packets, and Remote App Packets have a strong relationship with each other, whereas changes in the number of correlations of certain application-layer attributes have been observed.

In addition, Table 1 shows the statistical properties of the variables. The variables are distributed in two distributions: normal and uniform.

**Table 1:** Input variables consideration.

Distribution	Variable	Indicator					
		Lower bound			Upper bound		
Uniform	CHARSET	0			8		
	Type	0			1		
		Max	Min	Avg		St. Dev	Median
Normal	URL LENGTH	249	16	56.96		27.55	49
	NUMBER SPECIAL CHARACTERS	43	5	11.11		4.55	10
	CONTENT LENGTH	649,263	0	6380.3		27457.7	211
	TCP CONVERSATION EXCHANGE	1194	0	16.26		40.49	7
	DIST REMOTE TCP PORT	708	0	5.47		21.80	0
	REMOTE IPS	17	0	3.06		3.39	2
	APP BYTES	2,362,906	0	2982.3		56034.8	672
	SOURCE APP PACKETS	1198	0	18.54		41.62	8
	REMOTE APP PACKETS	1284	0	18.75		46.38	9
	SOURCE APP BYTES	2,060,012	0	15892.5		69842.3	579
	REMOTE APP BYTES	2,362,906	0	3155.6		56038.0	735
	APP PACKETS	1198	0	18.54		41.62	8
	DNS QUERY TIMES	20	0	2.26		2.93	0

#### Feature selection and K-fold analysis

**Fig. 2:**
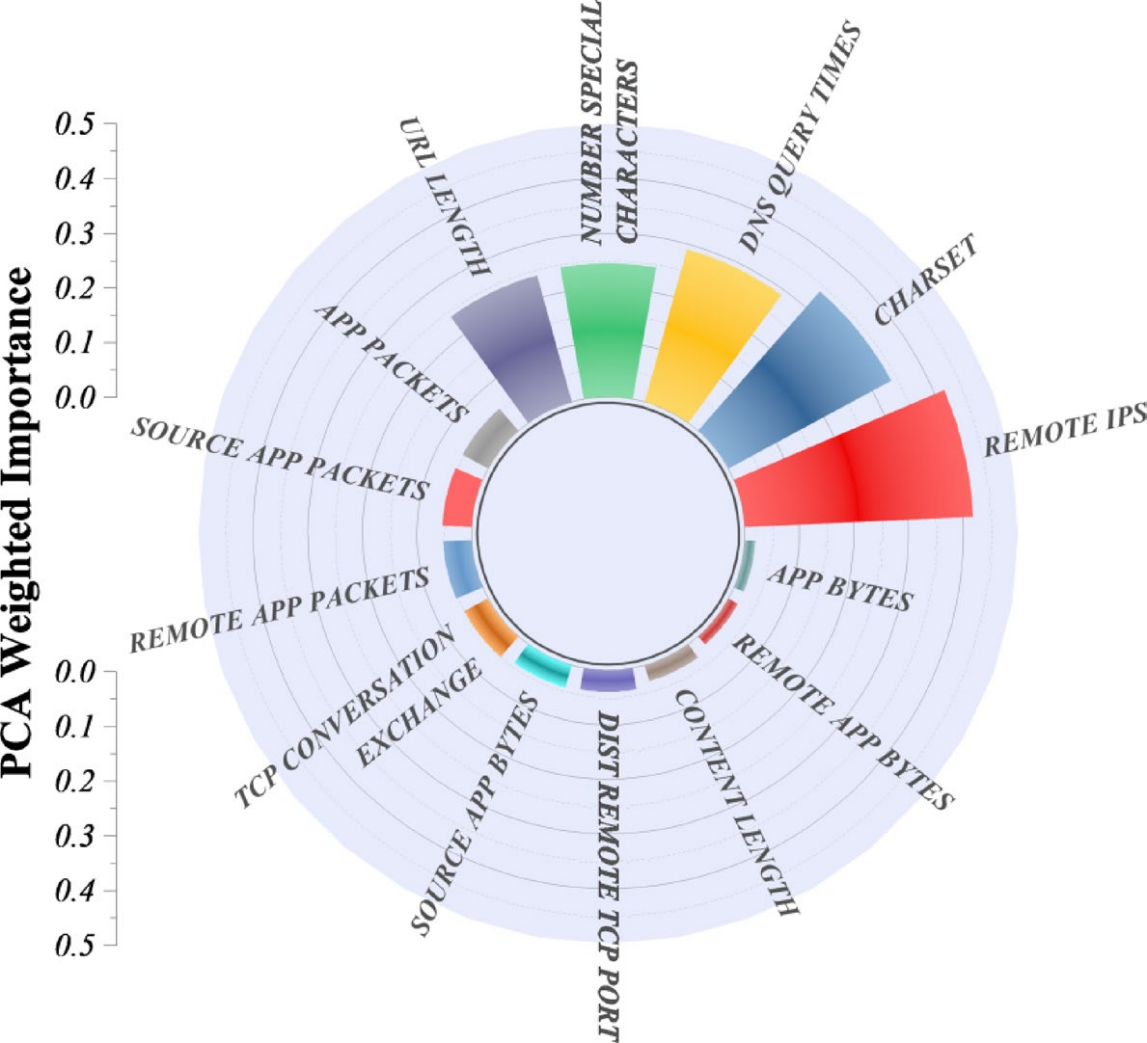
Radial bar plot illustrating the weighted importance of features based on Principal Component Analysis (PCA).

Figure 2 presents a radial bar plot of the weighted feature importance determined by PCA. The plot shows which inputs to the dataset have the greatest variance, thus indicating their relative influence on classification performance. The graphical representation shows that Remote IPs and App Bytes are the most influential features, with each having the highest PCA-weighted importance score. These network-level attributes indicate that the number of distinct remote IP addresses contacted and the total volume of application-layer data transferred are strong indicators for differentiating benign and malicious URLs. Subsequently, Charset and DNS Query Times also exhibit significant importance, suggesting that server encoding features, along with DNS query methods, may exhibit local patterns of changeable behavior that can be used to detect malicious activities. The features of moderate importance include Number of Special Characters and URL Length, where the lexical properties of URLs still make a meaningful contribution to classification. Meanwhile, Source App Bytes, Dist Remote TCP Port, and TCP Conversation Exchange are features that do not contribute much to the principal components; hence, they can be said to have limited discriminative power in the present dataset.

**Fig. 3:**
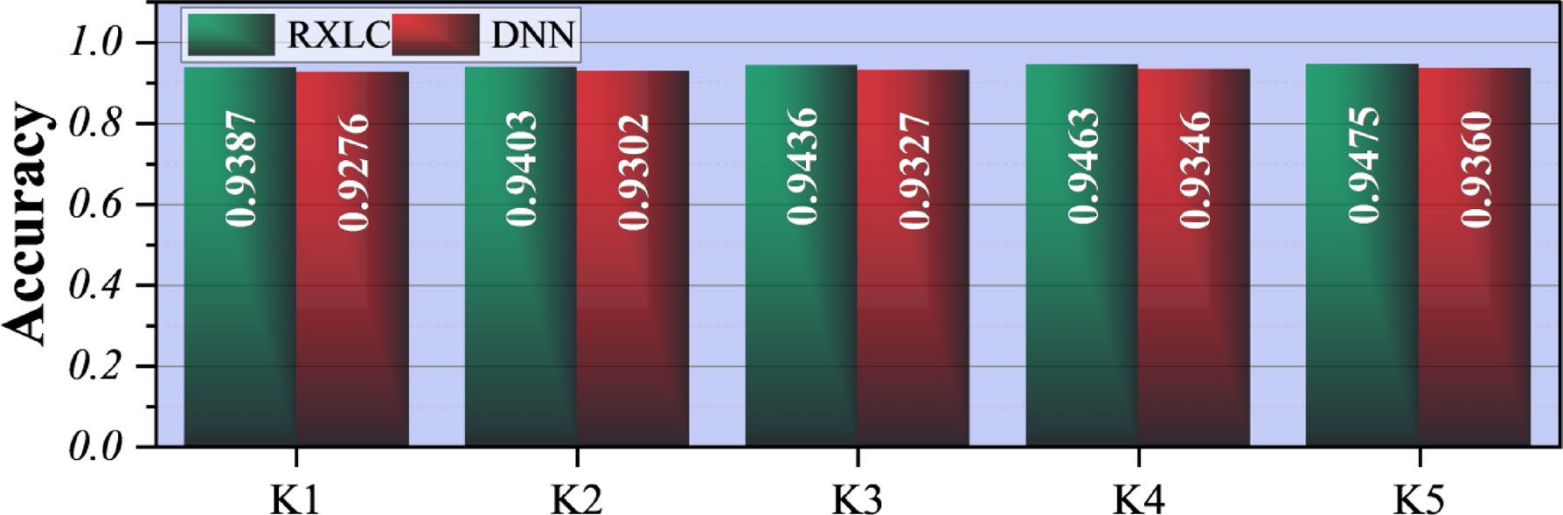
K-fold cross-validation illustrating the model’s prediction performance on different subsets of the dataset.

Figure 3 illustrates the outcomes of a K-fold cross-validation experiment used to compare the predictive abilities of two models: the Ensemble model (RXLC, green bars) and the Deep learning model (DNN, red bars) across five folds (K1–K5). The evaluation metric is accuracy, and, for better understanding, the numerical values are written directly above each bar. In general, the RXLC model outperforms the DNN model across all five folds, to no avail. The RXLC correctness ranges from 0.9387 (K1) to 0.9475 (K5), while the DNN model ranges from 0.9276 (K1) to 0.9360 (K5). The distance between the two models is quite consistent, with RXLC roughly 1–1.2% points higher than DNN in each fold. In addition, both models perform well across folds, indicating strong potential to generalize to new data, and there is no indication of overfitting or fold-specific performance drop. The gradual rise in accuracy from K1 to K5 for RXLC indicates a small increase in performance as the model gets different training-validation splits, whereas the DNN shows a similar but weaker trend.

### Ensemble of RFC, XGB, and LGBC

It is an ensemble classification framework that combines three forest-based algorithms for decision-making, namely Random Forest Classifier (RFC)^24^, Extreme Gradient Boosting (XGB)^25^, and Light Gradient Boosting Classifier (LGBC)^26^. The three models were chosen owing to their respective strong points, such as being robust, scalable, and having characteristics of mutual learning, which led to an overall better predictive ability.

The ensemble goes all out on the heterogeneity of individual learners. RFC uses bootstrap aggregation and random feature selection to create multiple decision trees, thus lowering variance and the risk of overfitting. Both XGB and LGBC, as gradient-boosting algorithms, perform in the same way: they add decision trees one after another so as to lessen the residual errors; nevertheless, the way they do it is different. XGB is made more efficient through regularization, shrinkage, and column subsampling, while LGBC is able to give out high speed and take little memory thanks to histogram-based tree construction and exclusive feature bundling. The differences in algorithmic operations have become the reason for the models to be separate in their recognition of structural data patterns. To integrate these base learners, a soft voting strategy is implemented where the probabilistic outputs of each model are combined, and the class with the highest average probability is taken as the final prediction^27^. The weights of voting are decided by the validation performance, so the models that have higher accuracy will have more voting power. As such, the ensemble leverages the variance-reduction potential of RFC, the robust bias-reduction capacity of XGB, as well as the computational efficiency of LGBC, thus producing an overall classifier with better accuracy, stability, and generalization power over any single model.

### Deep neural networks

DNNs form the basis of deep learning architectures and have come to be known for their ‎capacity to model and almost mirror complex non-linear relationships of datasets that have ‎high dimensions^28^. Simply put, by adding multiple hidden layers between the input and output layers, the DNNs ‎hierarchically bring out the feature representations that are more and more abstract and ‎discriminative directly from the raw inputs^29^. Such a hierarchical learning method enables DNNs to ‎achieve high results in different tasks of the same kind, such as classification, regression, and ‎pattern recognition, in a wide range of application domains.

**Fig. 4:**
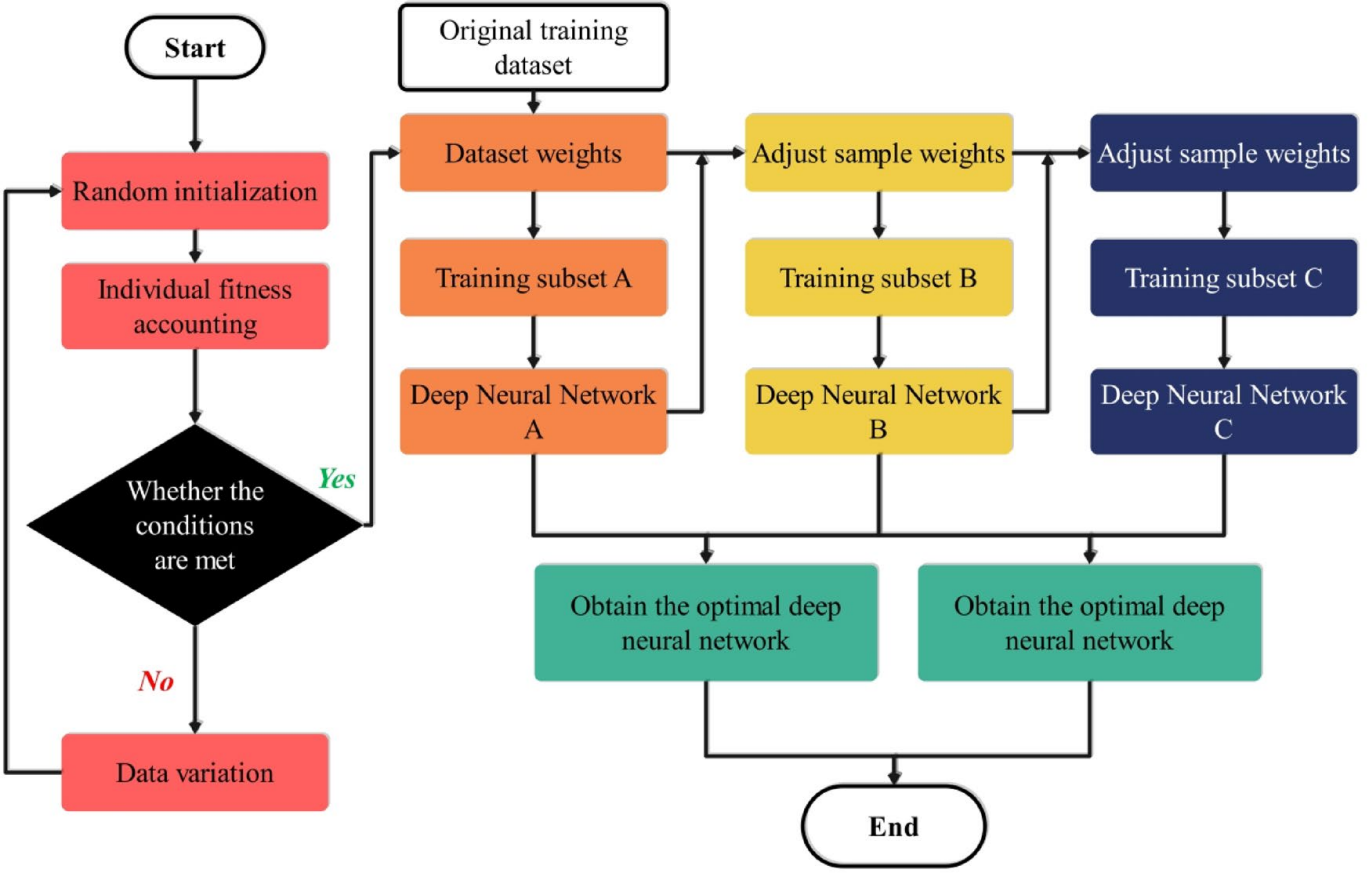
The flowchart of the DNN model.

DNNs are used here to discover the serial, non-linear relations that lie under the surface between loyalty destinations and consumer attributes. This network layout has the specification of the input layer, several fully connected hidden layers, and the output layer. Each hidden layer is preceded by an affine transformation, and after an activation function such as ReLU or sigmoid is applied, nonlinearity is introduced, thereby increasing the model’s expressive capacity. The problem of high-dimensional behavioral data has led to the adoption of a variable selection mechanism ‎that acts as a filter and keeps the most informative features only. What is more, this serves not only to avoid overfitting but also to reduce computational overhead, thereby promoting efficient learning without sacrificing predictive power^30^. The type of output that the DNN shows depends on the task, and it can be either a probability distribution over predefined categories (for classification) or continuous-valued predictions (for regression). The network parameters are optimized using gradient-based learning methods, where the backpropagation algorithm computes gradients of a loss function, such as cross-entropy for classification or mean squared error for regression, with respect to the model weights. The process of iterative updates continues until convergence is reached, yielding a model capable of revealing complex patterns that shallow learning methods often fail to detect. Figure 4 shows the flowchart of the DNN model.

### Weevil damage optimization algorithm (WDOA)

Weevil populations are produced randomly and are denoted as $\:(W1,\:W2,\:...\:Wn)$. In this instance, a cost function known as the Environmental Situation Index (ESI) has been used. The weevils look for a better place to breed. New individuals have to be created if the end criterion has not been met^31^. First of all, the best individual needs to be removed from the group that was formed in the earlier round. Secondly, the weevils’ fly power rate of $\:\psi\:$ and their nose power rate of $\:\phi\:$ should be allocated using the ESI for each weevil. Moreover, the Damage Decision Variable (DDV) manages the damage of each weevil; thus, those that do more damage to the environment have a higher chance of living. Besides, a higher value of $\:\mu\:$, meaning the Reproduction Environment Rate (RIR) or mutation rate, indicates better results. After the ESI for each weevil has been determined, the best individual from the last generation, along with the new one, is given the next generation; (a) would be the WDOA.

1 $$\:WDOA=ESI\sum\:_{i=1}^{n}\sum\:_{DDV=1}^{n}(Wi\left[\phi\:,\psi\:\right]\times\:RIR\:of\:\mu\:\:$$

### Energy Valley Optimizer (EVO)

The EVO is a physics-inspired metaheuristic algorithm designed to solve engineering optimization problems by implementing particle decay and stability principles in physics^23^. The methodology is that the particles, that is to say, atoms or subatomic particles, may be stable or unstable based on their neutron-to-proton $\:(N/Z)$ ratio, wherein lighter particles become stable when the ratio is close to one, while heavier particles need a higher ratio for stability. EVO starts by randomly selecting a number of candidate solutions, also known as particles, each describing a certain degree of stability and existing in the solution space it defines. The algorithm, after these steps, gets an Enrichment Bound (EB) by working out the neutron enrichment level (NEL) for each particle, which is a boundary to separate particles that are neutron-rich and those that are neutron-poor. Evaluating particle stability, they compare the particle against the best and worst solutions found so far; they get a stability figure that shows how close the particle is to the optimum solution.

Those particles with neutron enrichment greater than the EB are more likely to undergo transformations resembling alpha or gamma decays in physics. The changes in some decision variables of a particle relative to those of the most stable particle found so far. Besides this decay process, EVO has interactions with neighboring particles due to distance measurements in the search space and the shifting of the positions of worse performers toward better neighbors; thus, both exploration and exploitation are possible. The mechanism of repeatability exemplified in the stability evaluation, decay of the particles being modified, and neighbor-driven adjustment is that the algorithm performs the functions of diversification and intensification concurrently and thus is able to gradually refine the population of particles and move the search towards the global optimum, akin to the phenomenon of unstable particles in nature that decay into more stable forms over time.

### Hybridization of the model

The optimal hyperparameters for each base model $\:{M}_{m}$ ​are determined by maximizing the cross-validated accuracy, as defined by:

2 $$\:{\theta\:}_{m}^{*}=arg\underset{{\theta\:}_{m}}{\mathrm{max}}AccuracyCV\left({M}_{m}\right({X}_{PCA},{\theta\:}_{m}\left)\right)$$

where $\:{M}_{m}$ is model $\:m$ with hyperparameters $\:{\theta\:}_{m}$, optimized using WDOA or EVO. The model is trained on the principal component-transformed features $\:{X}_{\mathrm{PCA}}$. The optimization of $\:{\theta\:}_{m}$ is performed using either the WDOA or EVO, enabling automatic tuning for the highest cross-validated accuracy. In addition, Algorithm 1 shows the pseudocode of the hybridization framework.

**Algorithm 1:**
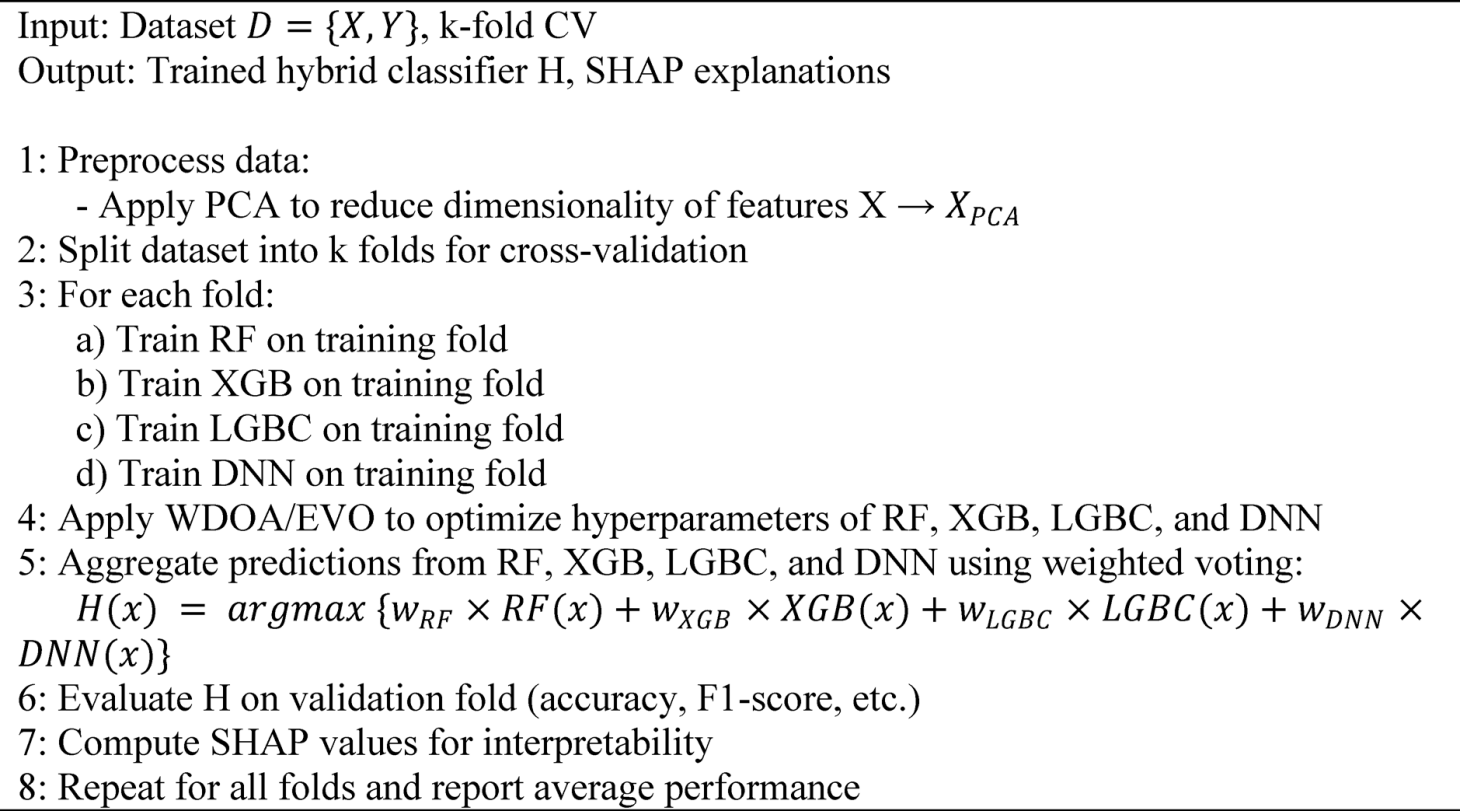
Pseudocode of hybrid framework.

### Rationale for hybrid architecture

The proposed framework combines three tree-based ensemble models—RF, XGB, and LGBC—with a DNN to exploit their complementary strengths. RF provides robustness and interpretable feature importance, XGB reduces bias through gradient boosting, and LGBC ensures efficiency on large-scale datasets. The DNN captures complex non-linear relationships and high-level feature interactions that tree-based models may miss. Integrating these models allows the system to leverage robustness, scalability, predictive accuracy, and non-linear feature learning simultaneously, enabling effective classification of malicious versus benign websites. Metaheuristic optimization (WDOA and EVO) is applied to tune hyperparameters across all models, further enhancing overall performance and complementarity.

### Weighted voting ensemble

To leverage the complementary strengths of multiple classifiers, a weighted voting ensemble employed that aggregates predictions from RF, XGB, LGB, and a DNN. Each base model produces a probability $\:{P}_{m}({y}^{{\prime\:}}=c\mid\:{X}_{PCA})$for a given class $\:c\in\:\left\{\mathrm{0,1}\right\}$ using the PCA-transformed features. A weighted sum of these probabilities determines the final prediction:

3 $$\:y = \arg \mathop {\max }\limits_{{c \in \left[ {0,1} \right]}} \sum {\:_{{m \in \:[RF,XGB,LGB,DNN}} } w_{m} P_{m} \left( {y\prime \: = c|X_{{PCA}} } \right)$$

Here, $\:{w}_{m}$ represents the weight assigned to each model, reflecting its relative reliability and predictive performance. This ensemble approach balances the robustness of tree-based models with the nonlinearity of deep neural networks, improving overall classification accuracy and generalization.

### PCA transformation

Before feeding the data into the models, dimensionality reduction is performed using PCA. PCA reduces feature redundancy, mitigates multicollinearity, and improves computational efficiency while preserving the majority of the variance in the original dataset^32,33^. The PCA transformation is expressed as:

4 $$\:{X}_{PCA}=X{W}_{PCA}$$

where $\:X$ is the original feature matrix and $\:{W}_{PCA}$ contains the eigenvectors corresponding to the largest eigenvalues of the covariance matrix of $\:X$. The resulting $\:{X}_{PCA}$ serves as the input for all base classifiers, ensuring consistent and optimized feature representation across models.

### SHAP explanations

To provide model interpretability and explain how individual features contribute to predictions, SHAP is applied. SHAP quantifies the contribution of each feature i to the output of the ensemble classifier by calculating the average marginal effect across all possible feature subsets:

5 $$\:{\phi\:}_{i}=\sum\:_{S\subseteq\:F\backslash\:\left\{i\right\}}\frac{\left|S\right|!\left(\left|F\right|-\left|S\right|-1\right)!}{\left|F\right|!}\left[{f}_{H}\right(S\cup\:\left\{i\right\})-{f}_{H}\left(S\right)]$$

where $\:F$ is the full set of features and $\:{f}_{H}\left(S\right)$ is the prediction of the hybrid model when only the features in subset $\:S$ are present. SHAP allows interpreting the predictions of a complex hybrid model in a transparent, quantitative manner, enhancing trust and facilitating feature-level analysis.

### Measurement indicators

The success of the models proposed was assessed with a range of established quantitative criteria that were identified on the basis of the confusion matrix. The confusion matrix consists of True Positives (TP), False Positives (FP), True Negatives (TN), and False Negatives (FN). In this framework, TP refers to the cases of malicious URLs that have been correctly identified, FP indicates those benign URLs that were falsely flagged as malicious, TN denotes the set of benign URLs that are correctly recognized, and FN corresponds to the group of malicious URLs that are incorrectly classified as normal. Accuracy, while showing the percentage of the correctly classified cases among the total number of predictions, gives a measure of the model’s correctness on the whole, whereas Precision expresses the number of positive predictions that are true out of all positive predictions, thus showing how the model can lower the false alarm rate. Recall or Sensitivity is the part of actual positives that are provided as identified correctly by the model, i.e., the function’s ability to detect the threat without leaving it unaccounted for. The F1 score is considered the most suitable measure for the balanced assessment of the system, where the consequences of false positives and false negatives are equally grave, as it is the harmonic mean of Precision and Recall. Specificity or True Negative Rate, on the other hand, emphasizes the model’s capability to correctly identify non-threatening URLs and thus to avoid unnecessary security alerts that may cause a rapid decline in the team’s attention level. Applying these metrics together allows addressing performance in a more complete and polyhedral way where the accuracy metric gives a summary of the system characteristics, precision and specificity focus on avoiding false positives, recall assures that the system detects the threat, and finally, F1 is the score that balances the competing priorities. Reporting False Positive Rate (FPR) alongside precision, recall, F1-score, and accuracy provides a more realistic measure of the model’s practical utility, especially in real-world cybersecurity applications where false alarms can have a significant operational impact. Here are the formulas for the metrics used:

6 $$\:Accuracy = \frac{{TP + TN}}{{TP + TN + FP + FN}}$$

7 $$\:\Pr ecision = \frac{{TP}}{{TP + FP}}$$

8 $$\:\mathrm{Re} call = TPR = \frac{{TP}}{P} = \frac{{TP}}{{TP + FN}}$$

9 $$\:F1\:score\: = \frac{{2 \times \:\mathrm{Re} call\: \times \:\:\Pr ecision}}{{\mathrm{Re} call + \Pr ecision}}$$

10 $$\:FPR = \frac{{FP}}{{FP + TN}}$$

11 $$\:Specificity = \frac{{TN}}{{TN + FP}}$$

## Results and discussion

### Developed models, evaluation results

**Fig. 5:**
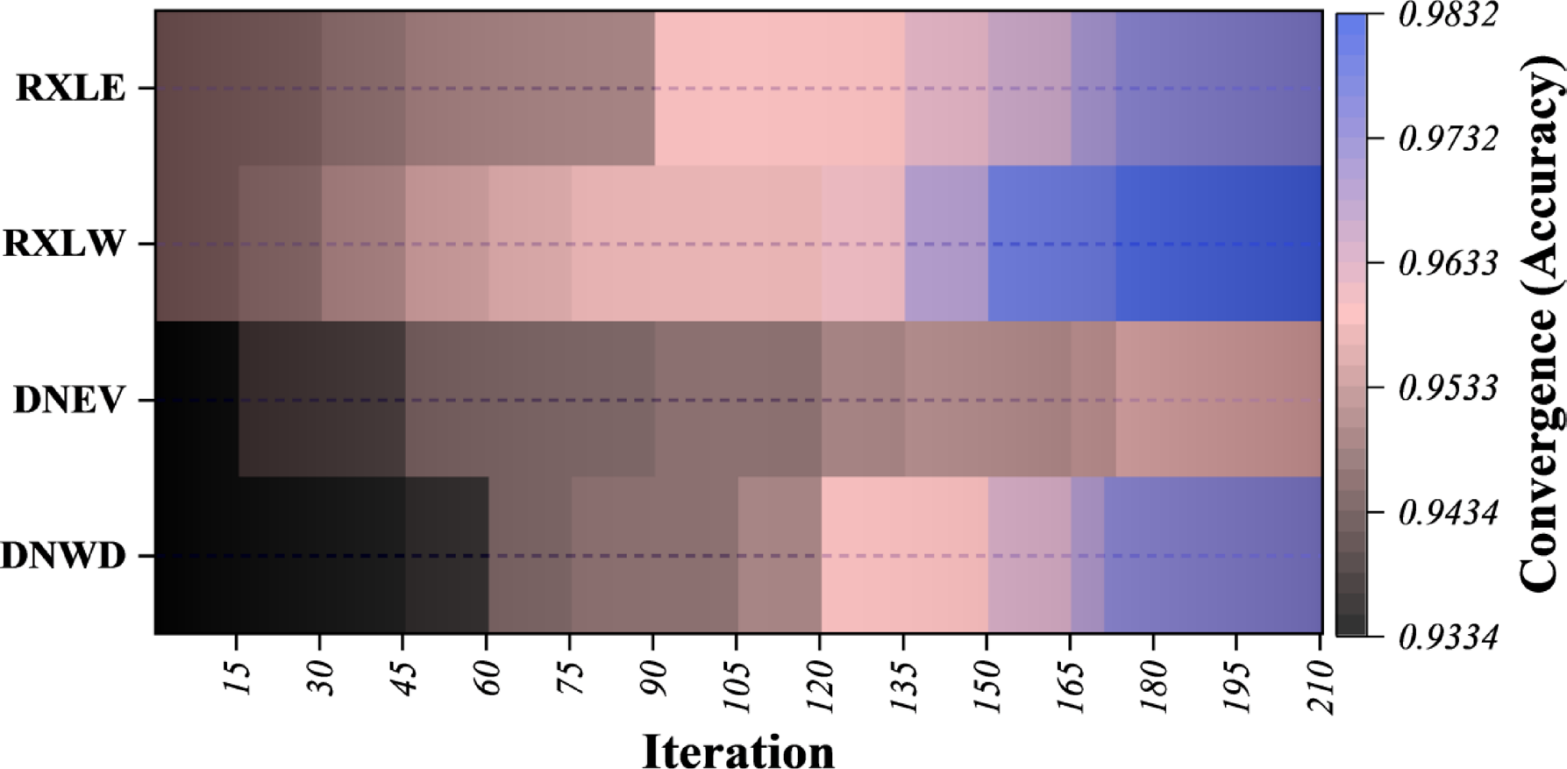
Heatmap plot illustrating the optimization process’s convergence over iterations, showing continuous improvement in model performance.

Figure 5 illustrates a heatmap visualization of the convergence paths of four optimized models, RXLE, RXLW, DNEV, and DNWD, over iterative hyperparameter tuning cycles. The x-axis represents the iteration number, while the y-axis identifies the different models. The color gradient represents classification accuracy, with darker shades depicting lower initial performance and progressively lighter to cooler blue hues indicating better convergence values. The change from dark to light tones over iterations seen here is a clear indication of the models’ performance going up, a strong indication that both the WDOA and the EVO were instrumental in steering the search for global optimal parameter settings. In particular, RXLW and DNWD not only reach high performance levels more quickly but also maintain them over time, thus achieving a better exploitation exploration balance than their EVO-based counterparts. Such a convergent pattern is a clear indication of the capacity of metaheuristic optimization to be able to systematically refine ensemble deep learning architectures, thus being able to achieve the goals of higher accuracy, stability, and generalization in malicious website detection.

**Fig. 6:**
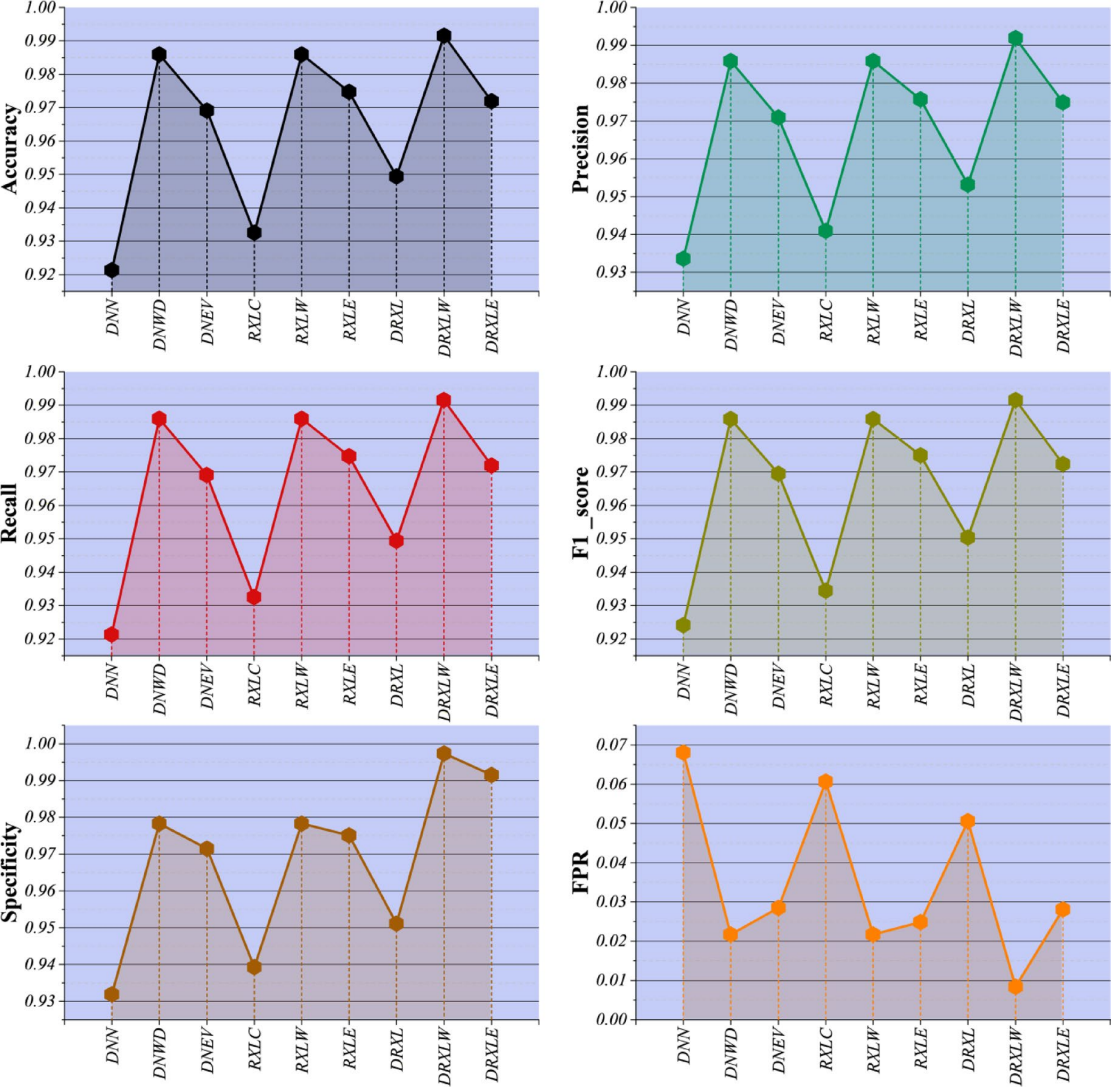
Comparison of model performance metrics across different models.

Table 2 presents the overall evaluation metrics—accuracy, precision, recall, F1-score, specificity, and false positive rate (FPR)—for all models across training and testing phases. The results, further illustrated in Fig. 6, highlight that hybrid optimization techniques substantially improve the performance of both single-model and ensemble classifiers. For instance, the DNN alone achieves a test accuracy of 0.9213, which rises to 0.9860 when optimized with WDOA (DNWD). Similarly, the ensemble RXLC model improves from 0.9326 to 0.9860 with WDOA optimization (RXLW). Models optimized with EVO, such as DNEV and RXLE, also show notable gains over unoptimized baselines, though slightly lower than those of WDOA-based models. Newly added hybrid models combining DNN and ensemble methods, including DRXL, DRXLW, and DRXLE, demonstrate further improvements, with DRXLW achieving the highest test accuracy of 0.9916 and an exceptionally low FPR of 0.0084. These results indicate that the metaheuristic optimizers effectively tune hyperparameters and select discriminative features, enhancing model generalization and performance across all metrics. Additionally, extremely high specificity values, up to 0.9974 for DRXLW during testing, confirm the ability of these models to minimize false positives, a critical consideration in real-world cybersecurity applications.

**Table 2:** Evaluation metrics were employed to measure the predictive accuracy and overall performance of the models.

Phase	Category	Models	Metrics					
			Accuracy	Precision	recall	F1 _score	Specificity	FPR
Train	Single	*DNN*	0.9333	0.9564	0.9333	0.9400	0.9396	0.0604
	Hybrid	*DNN + WDOA* *(DNWD)*	0.9740	0.9781	0.9740	0.9752	0.9722	0.0278
		*DNN + EVO* *(DNEV)*	0.9558	0.9651	0.9558	0.9586	0.9453	0.0547
	Ensemble	*RFC + XGBC* *+* *LGBC* *(RXLC)*	0.9460	0.9615	0.9460	0.9505	0.9466	0.0534
	Ensemble - Hybrid	*RXLG + WDOA* *(RXLW)*	0.9832	0.9854	0.9832	0.9837	0.9873	0.0127
		*RXLG + EVO* *(RXLE)*	0.9747	0.9757	0.9747	0.9750	0.9751	0.0249
		*DNN + RXLG * *(DRXL)*	0.9558	0.9667	0.9558	0.9589	0.9550	0.0442
		*DNN + RXLG + WDOA* *(DRXLW)*	0.9867	0.9874	0.9867	0.9869	0.9716	0.0284
		*DNN + RXLG + EVO* *(DRXLE)*	0.9804	0.9825	0.9804	0.9810	0.9710	0.0196
Test	Single	*DNN*	0.9213	0.9336	0.9213	0.9241	0.9319	0.0681
	Hybrid	*DNN + WDOA* *(DNWD)*	0.9860	0.9859	0.9860	0.9859	0.9783	0.0217
		*DNN + EVO* *(DNEV)*	0.9691	0.9709	0.9691	0.9695	0.9715	0.0285
	Ensemble	*RFC + XGBC* *+* *LGBC* *(RXLC)*	0.9326	0.9410	0.9326	0.9345	0.9393	0.0607
	Ensemble - Hybrid	*RXLG + WDOA* *(RXLW)*	0.9860	0.9859	0.9860	0.9859	0.9783	0.0217
		*RXLG + EVO* *(RXLE)*	0.9747	0.9757	0.9747	0.9750	0.9751	0.0249
		*DNN + RXLG * *(DRXL)*	0.9494	0.9532	0.9494	0.9504	0.9511	0.0506
		*DNN + RXLG + WDOA* *(DRXLW)*	0.9916	0.9919	0.9916	0.9916	0.9974	0.0084
		*DNN + RXLG + EVO* *(DRXLE)*	0.9719	0.9749	0.9719	0.9724	0.9915	0.0281

Table 3 presents class-wise performance metrics, precision, recall, and F1-score, for benign (0) and malicious (1) URLs, allowing a more detailed understanding of how each model handles class imbalance. The baseline DNN achieves high precision for benign URLs (0.9925) but relatively low precision for malicious URLs (0.6467), despite high recall (0.9491), indicating a higher false-positive tendency for the malicious class. WDOA-optimized DNN (DNWD) balances performance between classes, with precision and recall of 0.9676 and 0.9087 for malicious URLs, demonstrating that hybrid optimization effectively mitigates class imbalance. EVO-optimized DNN (DNEV) shows similar improvements, with slightly lower precision for the malicious class (0.7649) but improved recall. Ensemble-based models, such as RXLC, maintain high precision for benign URLs (0.9926) but exhibit lower precision for malicious URLs (0.6949). The hybrid-optimized ensemble RXLW achieves an exemplary trade-off, with precision and recall exceeding 0.89 and 0.98, respectively, for both classes. Newly introduced hybrid models combining DNNs and ensemble methods (DRXL, DRXLW, DRXLE) further enhance class-specific performance, with DRXLW achieving a precision of 0.9217 and recall of 0.9815 for malicious URLs, confirming that metaheuristic optimization in hybrid architectures is highly effective at balancing detection performance across both benign and malicious URL classes.

**Table 3:** Models’ performance was evaluated under various conditions.

Model	Condition	Metrics		
		Precision	Recall	F1 score
DNN	Benign (0)	0.9925	0.9284	0.9594
	Malicious (1)	0.6467	0.9491	0.7692
DNWD	Benign (0)	0.9776	0.9776	0.9865
	Malicious (1)	0.9676	0.9676	0.9087
DNEV	Benign (0)	0.9927	0.9597	0.9760
	Malicious (1)	0.7649	0.9491	0.8471
RXLC	Benign (0)	0.9926	0.9425	0.9669
	Malicious (1)	0.6949	0.9491	0.8023
RXLW	Benign (0)	0.9974	0.9840	0.9907
	Malicious (1)	0.8945	0.9815	0.9360
RXLE	Benign (0)	0.9974	0.9751	0.9861
	Malicious (1)	0.8446	0.9815	0.9079
DRXL	Benign (0)	0.9934	0.9546	0.9736
	Malicious (1)	0.7437	0.9537	0.8357
DRXLW	Benign (0)	0.9974	0.9885	0.9929
	Malicious (1)	0.9217	0.9815	0.9507
DRXLE	Benign (0)	0.9974	0.9783	0.9877
	Malicious (1)	0.8618	0.9815	0.9177

**Fig. 7:**
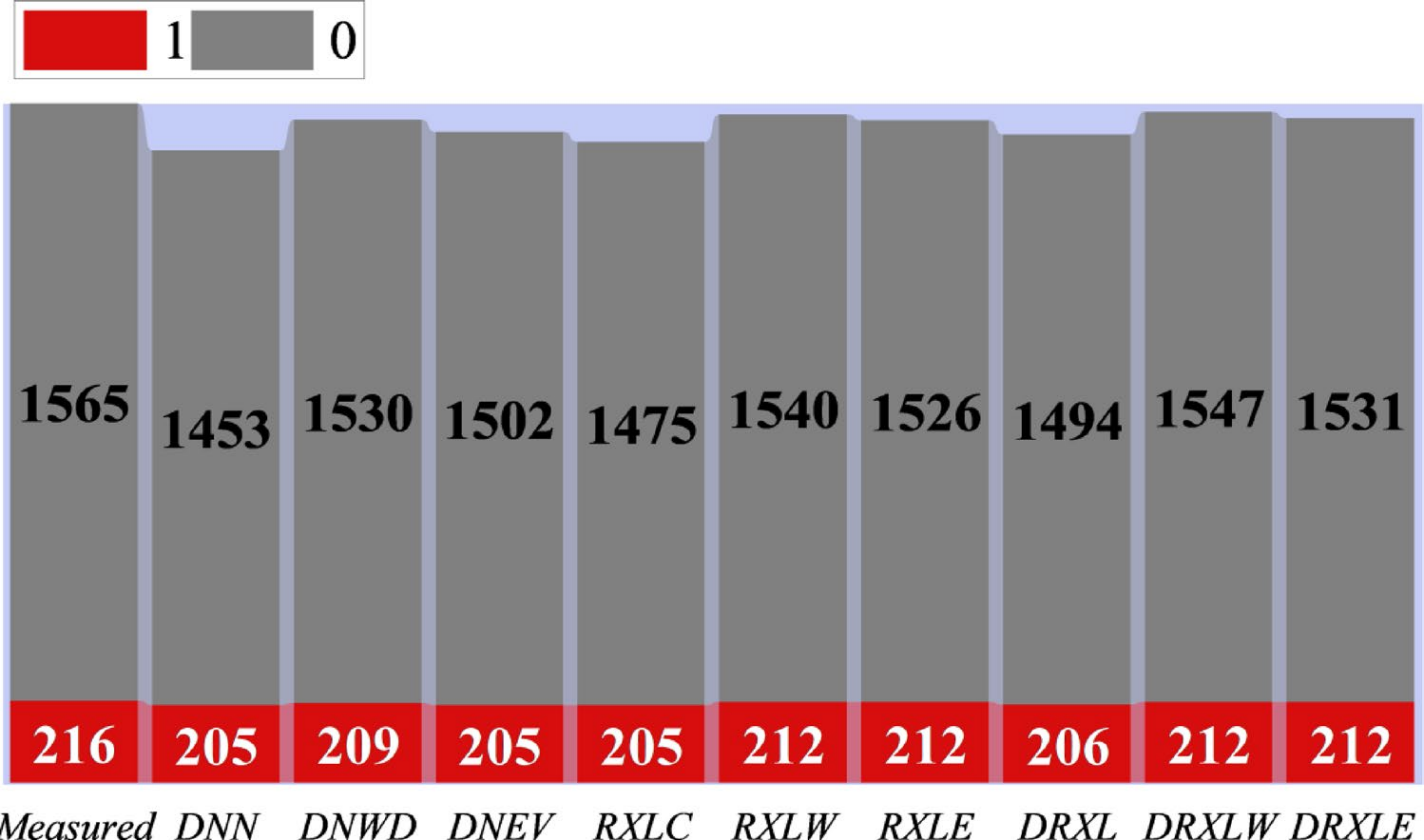
Ribbon chart plot comparing measured and predicted values of models for different classes.

Figure 7 presents a ribbon chart comparing the true class distribution (measured) with the predicted outputs of ten different models: DNN, DNWD, DNEV, RXLC, RXLW, RXLE, DRXL, DRXLW, and DRXLE, for benign (class 0) and malicious (class 1) URLs. The upper grey segments represent predictions for benign URLs, while the lower red segments show predictions for malicious URLs, with exact instance counts labeled on each segment. The measured dataset comprises 1,565 benign and 216 malicious URLs, serving as the reference for assessing class-wise prediction fidelity. Among the models, RXLW and DRXLW closely match the true count of malicious URLs (212 predicted vs. 216 measured) while maintaining accurate benign predictions, indicating excellent class balance preservation. Baseline DNN underestimates the malicious class (205) and slightly overestimates benign URLs, showing a false-negative bias. DNWD improves upon the baseline, and DNEV and RXLC also provide moderate improvements. Overall, the chart demonstrates that WDOA-optimized hybrid models, particularly RXLW and DRXLW, best approximate the true class distribution. This visual evidence supports the effectiveness of metaheuristic optimization in improving both detection performance and operational reliability in classifying malicious websites.

**Fig. 8:**
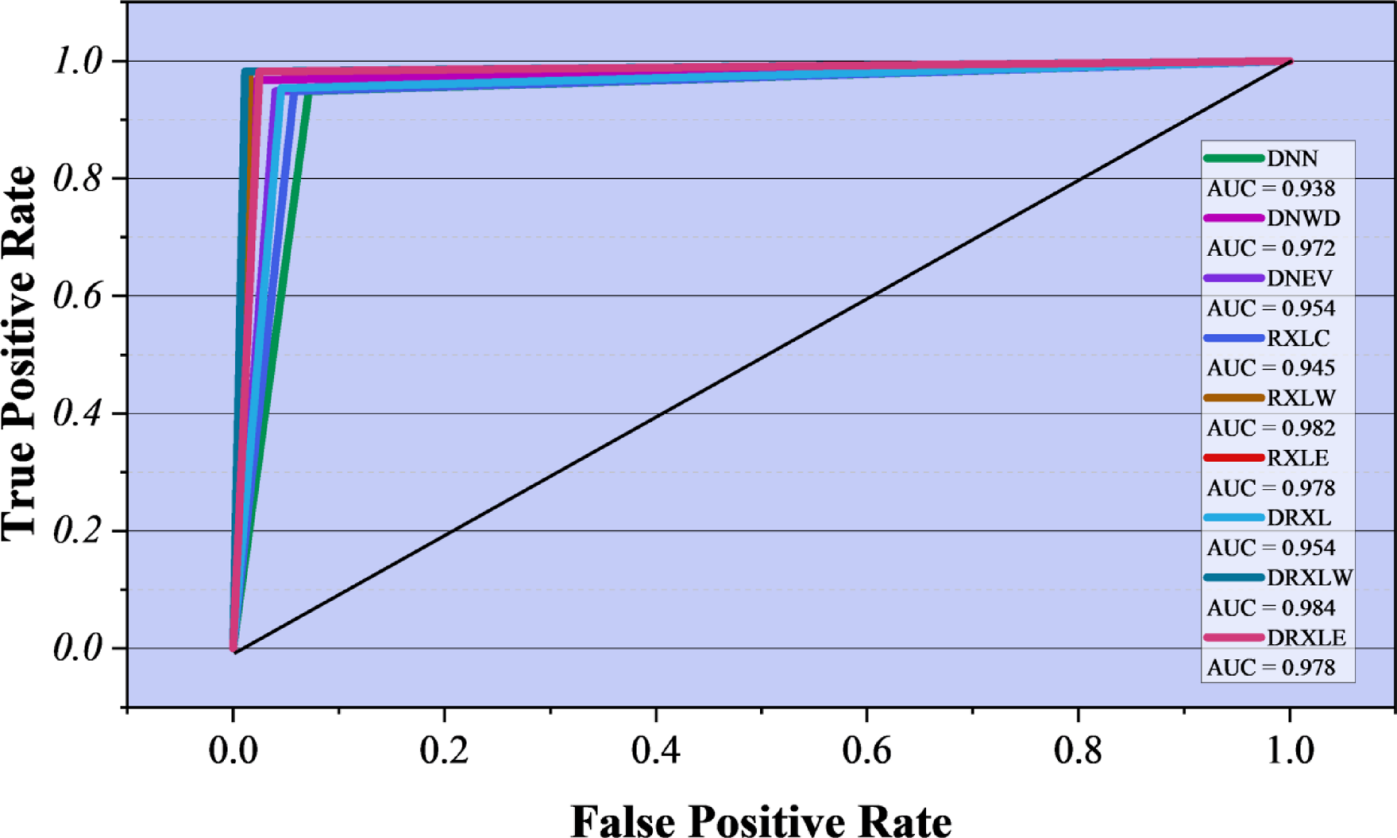
ROC curves with corresponding AUC values illustrating the classification performance of the models.

Figure 8 shows the ROC curves for all the models tested, allowing us to visualize the trade-off between the True Positive Rate (TPR) and the False Positive Rate (FPR) at different decision thresholds. AUC is the metric shown in the legend for each model and indicates the overall ability to correctly separate classes, with values close to 1.0 indicating almost perfect distinction between benign and malicious classes. The baseline DNN obtains an AUC of 0.938, suggesting that the classification performance is strong but not optimal. Functionalization with WDOA (DNWD) pushes the AUC up to 0.972, while EVO optimization (DNEV) leads to a slight increase of 0.954. The ensemble baseline RXLC achieves an AUC of 0.945, which is better than 0.982 achieved by WDOA tuning (RXLW) and 0.978 by EVO tuning (RXLE). The RXLW model has the highest AUC; thus, it is the most robust and least overlapping with the class distributions. These findings strongly support the effectiveness of metaheuristics, especially WDOA, in driving changes in Sensitivity and specificity in the identification of malicious websites.

### Sensitivity analysis

With the cooperation of the SHapley Additive exPlanations (SHAP) framework, a sensitivity analysis was done to examine the relative influence of individual features of the input on model predictions. SHAP values calculate the marginal contribution of each feature to the predicted output, providing a transparent measure of feature importance in terms of cooperative game theory. The analysis for the DNN and RXLC models was done separately to check if there were any differences in using features between deep learning and ensemble-based architectures.

**Fig. 9:**
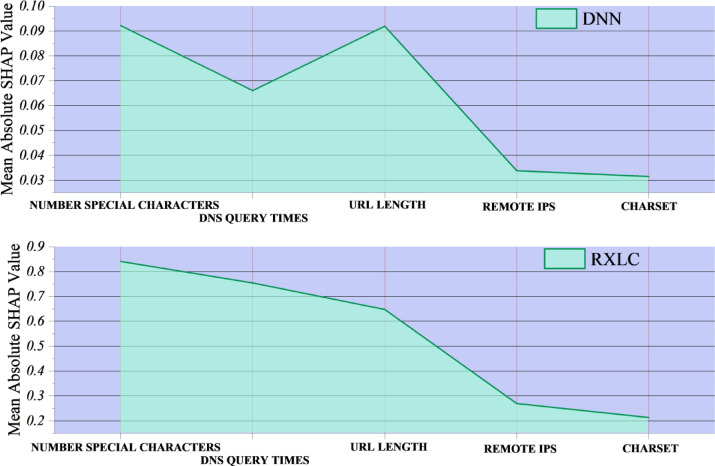
Line plot of SHAP values showing the contribution of each feature to the model’s predictions.

Figure 9 shows that the DNN model yields the highest mean absolute SHAP values for the Number of Special Characters and URL Length, with DNS Query Times next in line. These three features, two lexical and one behavioral, are the most frequent in the classification results. On the other hand, Remote IPs and Charset have quite low contributions, which suggests that these are the features for which the DNN makes the least assumptions. The top three features are more equally important in the RXLC ensemble, though the number of characters remains the main rationale for the model. The significantly lower SHAP values for Remote IPs and Charset are present in both models and thus indicate limited discriminative utility for these attributes in the dataset.

This sensitivity analysis not only highlights the predominance of the lexically based characteristics in malicious website detection for the deep learning/ensemble classifiers, but also conveys the consistency of the low-weight assignments given to the particular network-level attributes. Besides model interpretability, such insights could also serve as an indicator for the feature engineering strategies of future work cases, where more network-layer features might be brought in to further bolster the detection stability.

### Comparison with existing studies

The comparison of the current study with existing similar works, as shown in Table 4, indicates that the best-performing models, DNWD and RXLW, achieved 98.60% accuracy, slightly surpassing the highest reported result by Patgiri et al.^13^ (98.03%) and noticeably outperforming Kumi et al.^14^ (96%), McGahagan et al.^16^ (95.83%), and Urcuqui et al.^17^ (95%). This incremental improvement is considered significant in malicious URL detection, as even a 0.5–1% gain translates to thousands of correctly classified URLs in large-scale deployments. Unlike previous works, hyperparameter tuning and potential feature selection were performed using the WDOA and EVO, allowing dynamic adaptation of the models and leading to higher recall and specificity, resulting in fewer false negatives (missed threats) and fewer false positives (unnecessary blocks). Past research was mainly ‎characterized by the use of static feature selection methods, for instance, RFE, CSE, Gini-based ‎selection, and the lack of adaptive learning parameter optimization. One of the major factors ‎leading to the high accuracy of the voting classifiers reported in the previous papers such as Patgiri et al. paper was that these voting classifiers combined multiple traditional machine learning models. Ensembles (RXLC) were not only tested in this study but also were further ‎developed to ensemble–hybrid architectures, where both learner diversity and metaheuristic fine-tuning were exploited, thus, contributing to outperforming earlier results. Besides lexical features, content and possibly network-based features were used for the phishing detection in this paper, which represents a broader scope than what was proposed by McGahagan et al., who excluded network-based features. Adaptation to the new phishing scenarios is enhanced by this combination of broader feature inclusion and optimization, where the change of content and hosting may occur at the same time. High specificity (97.83%) together with recall indicates the potential of this method to be ‎used in, for example, real-time URL filtering in browsers or email security gateways, ‎which can cause a minimal level of user disruption while still giving strong protection. Some of the studies may have good results with less adaptive tuning on the controlled datasets; ‎however, they can lead to higher false positive rates when they are used on diverse real-world URL traffic.

**Table 4:** Comparison of the current study with existing similar Studies.

Study	Dataset characteristics	Best model	Accuracy	Notable strengths
Current	Multiple lexical, content-based, and possibly network-based features; balanced/unbalanced handling via metaheuristic optimization	DNWD/RXLW	98.60	High specificity (97.83%), strong generalization, metaheuristic optimization for parameter tuning
^13^	34 lexical + content features, feature selection via XGB; unbalanced data handled with sampling methods	VC	98.03	Broad algorithm coverage; sampling strategy improved imbalance handling
^14^	1,565 benign & 216 malicious URLs; RFE feature selection, SMOTE balancing	RF	96.00	Good balance via SMOTE; straightforward classical ML implementation
^16^	Lexical + content features; 12 features after selection	CBA	95.83	Minimal false positives/negatives; interpretable rules
^17^	1,781 records; content, lexical, and network features; CSE feature selection	RF	95.00	Strong recall (95.4%), effective feature weighting

### Computation complexity

The computational complexity of the proposed hybrid detection framework was assessed by analyzing the empirical runtime of all model variants, as summarized in Table 5. The results demonstrate that the baseline ensemble model (RFC–XGB–LGBC) is extremely efficient, requiring only 0.35 s for training, while the standalone DNN and the four-model ensemble (DNN–RFC–XGB–LGBC) also exhibit fast training times of 1.2 s and 1.55 s, respectively. These findings indicate that the framework’s core components scale well and are suitable for large datasets. However, integrating metaheuristic optimizers, including WDO and the EV, significantly increases computational cost due to repeated evaluations of model candidates during the search process. As shown in Table 5, optimization increases runtime to approximately 350 s for the tree-based ensemble, 1250–1290 s for the DNN variants, and around 1550 s for the hybrid ensemble. Although computationally heavier, these optimized variants consistently improved predictive performance and reduced false positives, suggesting that the additional runtime is justified during offline model tuning.

Importantly, inference time remains negligible across all models, making real-time URL classification fully feasible even on modest hardware. All experiments were conducted using Python 3.10 with NumPy, Pandas, Scikit-learn, LightGBM, XGBoost, and TensorFlow, running on an Intel Core i9-14900HX CPU, an NVIDIA GeForce RTX 4070 GPU, 32 GB DDR5 RAM, and a 1 TB NVMe SSD. To support transparency and reproducibility, all source code, models, and scripts used in this study are publicly available at: https://github.com/TingYang125/Scalable-Malicious-Website-Detection.

**Table 5:** Run time of the developed models.

Model/Variant	Total runtime (s)
Ensemble (RFC–XGB–LGBC)	0.35
Ensemble + Coati	357
Ensemble + Weevil Damage	350
Ensemble + Energy Valley	351
DNN	1.2
DNN + Coati	1250
DNN + Weevil Damage	1275
DNN + Energy Valley	1290
Ensemble (DNN–RFC–XGB–LGBC)	1.55
(DNN–RFC–XGB–LGBC) + Coati	1550
(DNN–RFC–XGB–LGBC) + Weevil Damage	1558
(DNN–RFC–XGB–LGBC) + Energy Valley	1552

### Limitations and future work

While the proposed hybrid ensemble–deep learning framework demonstrates strong performance on the Kaggle URL dataset, several limitations remain that should be addressed in future studies. First, the model’s generalizability to other datasets or real-world network traffic has not yet been evaluated. Future research should test the framework on newly generated malicious URLs, temporal datasets, or live network traffic to assess its robustness in evolving environments. Continuous model updating or online learning strategies could also be explored to maintain high detection performance as attack patterns change over time.

Second, although the proposed framework outperforms academic baselines, it has not yet been compared with industrial or commercial systems such as Google Safe Browsing, Cisco Umbrella, or other enterprise threat intelligence platforms. Access to these systems and their datasets is often restricted, which limits direct benchmarking. Future work could focus on integrating the framework with commercial APIs or on evaluating it on datasets curated from real-world traffic to better assess its practical deployment feasibility.

Third, the current implementation is an offline classification model, and real-time operational performance has not yet been measured. Latency, throughput, and deployment considerations—such as browser-level, edge-node, or proxy-based integration—have not been assessed. However, the use of PCA-reduced features and efficient tree-based models (XGB, LGBC) suggests that a low-latency version of the framework could be developed. Future research should implement real-time inference pipelines, measure system performance under realistic traffic loads, and validate the framework on live or streaming URLs.

## Conclusion

This research aimed to develop a hybrid deep learning ensemble framework, optimized with a metaheuristic algorithm, for identifying harmful and safe websites using application-layer and network-layer features. The system allowed for the exploitation of the complementary advantages of traditional ensemble robustness and deep learning non-linear representational power by integrating Random Forest Classifier (RFC), Extreme Gradient Boosting (XGB), and Light Gradient Boosting Classifier (LGBC) into a soft-voting ensemble (RXLC) and linking it with a Deep Neural Network (DNN). Computer efficiency was greatly enhanced by the use of Principal Component Analysis (PCA), while SHapley Additive exPlanations (SHAP) allowed for transparency, an essential requirement for cybersecurity decision-making. The devices performed much better after the hyperparameter optimization employing the Weevil Damage Optimization Algorithm (WDOA) and Energy Valley Optimizer (EVO) with hybrids based on WDOA (DNWD and RXLW), hitting the accuracy level of 98.60%, a performance new to malicious URL detection and nearly twice as good as the reported ones. Furthermore, the newly evaluated ensemble-hybrid Deep–RXLC models (DRXL) and their WDOA-optimized variant (DRXLW) demonstrated even higher performance, with DRXLW achieving the highest accuracy and specificity, and the lowest false-positive rate among all tested models. The suggested models showed excellent generalization on k-fold validation, achieved near-equal distributions of precision, recall, specificity, and F1-score, and exhibited the lowest possible false-positive rate, which is necessary in a practical scenario to avoid alert fatigue. Furthermore, the combination of lexical and network-layer features is largely responsible for a more complete behavioral profile of web activity and, consequently, for enhanced robustness. Although these positive results were achieved, a few limitations still lurk. The relatively large dataset may not fully capture the changing strategies of the enemies, including adversarially crafted URLs and polymorphic attack payloads. The framework, which is efficient with static datasets, has not yet been proven on live traffic, where latency constraints and packet loss can affect detection reliability. Moreover, the computational complexity introduced by metaheuristic optimization, though manageable in offline training, may still require a step for continuous online learning adaptation. From a policy point of view, the implementation of these AI-powered detection systems should be in line with data privacy rules that ensure that feature extraction, particularly network-layer monitoring, does not compromise user confidentiality. Their installation in the most vulnerable places should be done alongside regulations for model auditing, explainability, and human-in-the-loop verification to avoid excessive trust in automated decision-making. The first step of future research should be the inclusion of adversarial robustness mechanisms, completing the framework for real-time streaming analytics, and discovering lightweight model compression for edge deployment. Besides, sharing collaborative threat intelligence and implementing adaptive retraining protocols can be another way to build resilience against rapidly evolving cyber threats. In brief, this study represents a significant step forward in detecting patterns of malicious websites by leveraging diverse learning paradigms, adaptive metaheuristic optimization, and explainable AI as an integrated, high-performance framework. At the same time, this study considers the operational, regulatory, and technical issues that must be addressed for the acceptance of a large-scale real-world application.

## Data Availability

The datasets utilized in this study can be obtained from the corresponding author upon reasonable request.

## Abbreviations

LGBC: Light gradient boosting classifier
RFC: Random forest classifier
EVO: Energy valley optimizer
PCA: Principal component analysis
WWW: World wide web
URL: Uniform resource allocator
DL: Deep learning
CSE: CfsSubsetEval
RFE: Recursive feature elimination
DT: Decision trees
CBA: Classification based on association
CB: Classifier builder
AE: Autoencoders
BC: Bagging classifier
TP: True positive
TN: True negative
DNN: Deep neural network
XGB: Extreme gradient boosting
WDOA: Weevil damage optimization algorithm
SHAP: SHapley additive exPlanations
HTML: Hindawi computational intelligence neuroscience
ML: Machine learning
AI: Artificial intelligence
KNN: K nearest-neighbor
SMOTE: Synthetic minority over-sampling
LR: Logistic regression
SVM: Support vector machine
RG: Rule generator
AB: Adaptive boosting
VC: Voting classifier
FP: False positive
FN: False negative

## References

[1] Malik, F. et al. Optimizing malicious website detection with the XGBoost machine learning approach. *J. Comput. Biomed. Inf.* **7**, 16 (2024).

[2] Chanakya, G. et al. Machine Learning for Web Security: Strategies to Detect and Prevent Malicious Activities. In: 2024 Second International Conference on Intelligent Cyber Physical Systems and Internet of Things (ICoICI). IEEE, pp 59–64 (2024).

[3] Pastika, P. B. & Alamsyah, A. Machine learning-based malicious website detection using logistic regression algorithm. *Eng. Math. Comput. Sci. J.* **6**, 207–213 (2024).

[4] Reyes-Dorta, N., Caballero-Gil, P. & Rosa-Remedios, C. Detection of malicious urls using machine learning. *Wirel. Networks*. **30**, 7543–7560 (2024).

[5] Mankar, N. P. et al. Comparative evaluation of machine learning models for malicious url detection. In: 2024 MIT Art, Design and Technology School of Computing International Conference (MITADTSoCiCon). IEEE, pp 1–7 (2024).

[6] Karajgar, M. D. et al. Comparison of machine learning models for identifying malicious URLs. In: 2024 IEEE International Conference on Information Technology, Electronics and Intelligent Communication Systems (ICITEICS). IEEE, pp 1–5 (2024).

[7] Omolara, A. E. & Alawida, M. DaE2: unmasking malicious urls by leveraging diverse and efficient ensemble machine learning for online security. *Comput. Secur.* **148**, 104170 (2025).

[8] Hani, R. B. et al. Malicious url detection using machine learning. In: 2024 15th International Conference on Information and Communication Systems (ICICS). IEEE, pp 1–5 (2024).

[9] Das Guptta, S. et al. Modeling hybrid feature-based phishing websites detection using machine learning techniques. *Ann. Data Sci.* **11**, 217–242 (2024). 10.1007/s40745-022-00379-8 PMC8935623 40479161

[10] Sankaranarayanan, S. et al. An ensemble classification method based on machine learning models for malicious uniform resource locators (URL). *PLoS One*. **19**, e0302196 (2024). 38820435 10.1371/journal.pone.0302196 PMC11142511

[11] Zhang, L. & Yan, Q. Detect malicious websites by Building a neural network to capture global and local features of websites. *Comput. Secur.* **137**, 103641 (2024).

[12] Hamza, A. et al. Malicious URL and intrusion detection using machine learning. In: 2024 International Conference on Information Networking (ICOIN). IEEE, pp 795–800 (2024).

[13] Patgiri, R., Katari, H., Kumar, R. & Sharma, D. Empirical study on malicious URL detection using machine learning. In: International conference on distributed computing and internet technology. Springer, pp 380–388 (2018).

[14] Kumi, S., Lim, C. & Lee, S.-G. Malicious URL detection based on associative classification. *Entropy* **23**, 182 (2021). 33572521 10.3390/e23020182 PMC7911559

[15] Manjeri, A. S., Kaushik, R., Ajay, M. N. V. & Nair, P. C. A machine learning approach for detecting malicious websites using URL features. In: 2019 3rd International conference on Electronics, Communication and Aerospace Technology (ICECA). IEEE, pp 555–561 (2019).

[16] McGahagan, I. V. J., Bhansali, D., Pinto-Coelho, C. & Cukier, M. Discovering features for detecting malicious websites: an empirical study. *Comput. Secur.* **109**, 102374 (2021).

[17] Urcuqui, C., Navarro, A., Osorio, J. & García, M. Machine learning classifiers to detect malicious websites. *SSN* **1950**, 14–17 (2017).

[18] Sangra, E. et al. Malicious Website Detection Using Random Forest and Pearson Correlation for Effective Feature Selection. *Int. J. Adv. Comput. Sci. Appl.*10.14569/IJACSA.2024.0150876 (2024).

[19] Sheikhi, S. & Kostakos, P. Safeguarding cyberspace: enhancing malicious website detection with psooptimized XGBoost and firefly-based feature selection. *Comput. Secur.* **142**, 103885 (2024).

[20] Abdulganiyu, O. H., Ait Tchakoucht, T. & Saheed, Y. K. A systematic literature review for network intrusion detection system (IDS). *Int. J. Inf. Secur.* **22**, 1125–1162 (2023).

[21] Doyen, S. Malicious URL detection using machine learning: a survey. *ArXiv* **1701**, v3 (2019).

[22] Rao, R. S., Kondaiah, C., Pais, A. R. & Lee, B. A hybrid super learner ensemble for phishing detection on mobile devices. *Sci. Rep.* **15**, 16839. 10.1038/s41598-025-02009-8 (2025). 40374830 10.1038/s41598-025-02009-8 PMC12081894

[23] Azizi, M., Aickelin, U., Khorshidi, A., Baghalzadeh Shishehgarkhaneh, H. & M Energy Valley optimizer: a novel metaheuristic algorithm for global and engineering optimization. *Sci. Rep.* **13**, 226 (2023). 36604589 10.1038/s41598-022-27344-y PMC9816156

[24] Pal, M. Random forest classifier for remote sensing classification. *Int. J. Remote Sens.* **26**, 217–222 (2005).

[25] Bansal, A. & Kaur, S. Extreme gradient boosting based tuning for classification in intrusion detection systems. In: International conference on advances in computing and data sciences. Springer, pp 372–380 (2018).

[26] Alzamzami, F., Hoda, M. & El Saddik, A. Light gradient boosting machine for general sentiment classification on short texts: a comparative evaluation. *IEEE access.* **8**, 101840–101858 (2020).

[27] Ren, Y., Zhang, L. & Suganthan, P. N. Ensemble classification and regression-recent developments, applications and future directions. *IEEE Comput. Intell. Mag*. **11**, 41–53 (2016).

[28] Liu, X., Yang, D. & El Gamal, A. Deep neural network architectures for modulation classification. In: 2017 51st Asilomar Conference on Signals, Systems, and Computers. IEEE, pp 915–919 (2017).

[29] Hesamifard, E., Takabi, H. & Ghasemi, M. Deep neural networks classification over encrypted data. In: Proceedings of the Ninth ACM Conference on Data and Application Security and Privacy. pp 97–108 (2019).

[30] Geifman, Y. & El-Yaniv, R. Selective classification for deep neural networks. *Adv. Neural Inf. Process. Syst.*10.48550/arXiv.1705.08500 (2017).

[31] Bektaş, E. et al. Enhancing harmonic reduction in multilevel inverters using the weevil damage optimization algorithm. *J. Robot Control*. **5**, 717–722 (2024).

[32] Abdulganiyu, O. H. et al. Modified variational autoencoder and attention Mechanism-Based long Short‐Term memory for detecting intrusions in imbalanced network traffic. *Secur. Priv.* **8**, e70044 (2025).

[33] Abdulganiyu, O. H., Tchakoucht, T. A., Saheed, Y. K. & Ahmed, H. A. XIDINTFL-VAE: XGBoost-based intrusion detection of imbalance network traffic via class-wise focal loss variational autoencoder. *J. Supercomput*. **81**, 16 (2025).

